# Editorial: Full landscape of human genomic diversity and its impact on precision medicine

**DOI:** 10.3389/fgene.2024.1432509

**Published:** 2024-06-14

**Authors:** Guanglin He, Hui-Yuan Yeh, Mengge Wang

**Affiliations:** ^1^ Center for Archaeological Science, Sichuan University, Chengdu, China; ^2^ Institute of Rare Diseases, West China Hospital of Sichuan University, Sichuan University, Chengdu, China; ^3^ School of Humanities, Nanyang Technological University, Singapore, Singapore

**Keywords:** genomic diversity, fine-scale population substructure, genome-wide SNP, paternal structure, maternal lineages

## Introduction

Directing genetic analysis towards ethnolinguistically diverse populations presents a unique opportunity to comprehensively explore human genetic diversity, trace the evolutionary paths of distinct populations, and understand how specific genetic backgrounds influence disease susceptibility and complex traits. However, the current focus of large-scale genetic research on European populations is limiting our understanding (Bick et al., 2024). The underrepresentation of non-European populations in these studies not only hampers the development of disease risk models but also prevents us from fully capturing the vast spectrum of human genetic diversity (Sun et al., 2023; He et al., 2024; Li et al., 2024; Sun et al., 2024). This is because demographic history, linkage disequilibrium, and adaptive evolutionary histories vary significantly across different continental groups.

While the first human genome was sequenced two decades ago and its reference coordinates were made publicly available, the insights gleaned from the Human Genetic Diversity Project (HGDP) and the 1000 Genomes Project (1KGP) have only scratched the surface of the origins, migrations, diversity, and evolutionary histories of underrepresented populations (Bergström et al., 2020; Byrska-Bishop et al., 2022). Recent genomic projects like United Kingdom Biobank 100 K and TopMed have certainly advanced our understanding of the genetic bases of human diseases and complex traits (Taliun et al., 2021; Rubinacci et al., 2023). However, to truly grasp the genetic similarities and differences among continental populations from both evolutionary and medical perspectives, we need to embark on further studies, such as the 10K Chinese People Genomic Diversity Project (10K_CPGDP) (He et al., 2023).

### Population stratification inferred from autosomal genomic legacy

Ancient DNA work based on autosomal variations has illuminated extensive population migrations and admixtures of spatiotemporally different populations that reshaped ancient and modern human populations (Mallick et al., 2024). Wang et al. conducted genome-wide single nucleotide polymorphisms (SNPs) genotyping on one Tibeto-Burman (TB), one Hmong-Mien (HM), and two Tai-Kadai (TK) groups in Guizhou Province, integrating this new data with existing datasets from 16 linguistically/geographically proximate Guizhou populations and 218 ancient and modern East Asian groups for a comprehensive demographic analysis. This study revealed language-related population stratification among Chinese populations and identified a unique HM-related genetic lineage. Admixture model reconstructions and admixture timing estimates support the hypothesis that HM populations originated from the Yungui Plateau and migrated southward historically. Additionally, despite the proximity, the TK and TB groups in Guizhou exhibited distinct population structures from HM groups but showed significant gene flow between them. Feng et al. utilized short tandem repeats (STRs) genotyping on 628 Hakka individuals from Guangdong province using the Forensic Analysis System Multiplecues SetB Kit, providing insights into the genetic diversity of southeastern China’s Indigenous populations. The study highlighted that clustering patterns based on length polymorphisms significantly differ from those based on sequence polymorphisms, suggesting that relying solely on length polymorphisms may overlook detailed genetic architectures of ethnolinguistically diverse Chinese populations. These findings advance our understanding of the genetic diversity and demographic history of Chinese populations from an autosomal perspective.

### Uniparental genetic evidence for fine-scale evolutionary history reconstruction

Genetic variations in the non-recombining portion of the Y-chromosome (NRY) and mitochondrial DNA (mtDNA) offer unique insights into human evolutionary history due to their haploid inheritance. Li et al. examined the maternal genetic landscape of highland Tibetans by analyzing the complete mitochondrial genomes of 145 native individuals from Lhasa. Common maternal lineages among these Tibetans included M9a, R, F1, D4, N, and M62, with Tibetan-specific lineages such as A11, A21, M9, M13, and M62 also identified. The distribution and clustering of haplogroups among geographically diverse Tibetan groups and East Asian reference populations indicated that multiple admixture events between lowland and highland populations across various historical periods have significantly influenced the matrilineal genetic structure of highland Tibetans.

Geography and language-related substructures among different populations were reflected in Y-chromosome variations (Wang et al., 2023). Wang et al. conducted genotyping of 110 Y-SNPs on 209 Altay Kazakhs and 201 Ili Kazakhs in Xinjiang and performed a comprehensive analysis by integrating the previously reported data of 24 Y-STRs. They found that the paternal lineages of Altay Kazakhs showed greater diversity than those of other geographically diverse Kazakhs. The network-based topology of C2a1a3-F1918 dominant among newly genotyped Kazakhs revealed that Altay Kazakhs derived most of their paternal lineages from Kerey-Abakh ancestry, and Ili Kazakhs derived most of their paternal lineages from Kerey-Ashmaily ancestry. The TMRCA estimation (289.4 ± 202.65 years) of the DYS448-23 subcluster belonging to C2a1a3-F1918 and the patterns of haplogroup distribution confirmed the northeast Asian origin of Xinjiang Kazakhs and genetic influence from the 18th-century expansion of the Kerey clan. Yu et al. generated 100 new sequences of C2a-M48-SK1061 and jointly analyzed 140 published sequences belonging to this lineage to reconstruct a highly revised phylogenetic tree and estimate the TMRCA of different subclades. They identified several sublineages almost unique to Ewenki, Evens, Oroqen, Xibe, Manchu, Daur, and Mongolian people. The revised phylogeny provided a clear picture of the phylogenetic history of C2a-M48-SK1061 over the last 2000 years. Yu et al. also analyzed 229 sequences belonging to N1a2a-F1101, constructed a highly revised phylogeny containing age estimates of N1a2a-F1101, and explored the patterns of geographical distribution of the N1a2a-F1101 sublineage. The initial differentiation location and estimated expansion times of N1a2a-F1101 suggested that the expansion of the Bronze Age people in the border areas of the eastern Eurasian steppe and North China not only played a crucial role in the development of early Chinese states and civilizations but also left essential traces in the gene pool of genetically distinct Chinese populations. To deeply explore the fine-scale patrilineal genetic structure of Xinjiang Mongolians, Wang et al. genotyped 165 Xinjiang Mongolian males using 108 Y-SNPs and 44 Y-STRs. They found that four paternal lineages, C2a1a3-F1918, C2a1a2-M48, N1a1a-M178, and R1a1a-M17, occurred frequently among Xinjiang Mongolians. Integrated analysis of ancient genomes and newly generated Y-chromosome sequences, as well as the TMRCA estimates of R1a1a-M17, indicated that one of the Xinjiang Mongolian-related ancestral lineages came from Northeast Asia, and they subsequently mixed with local Xinjiang populations.

## Conclusion and perspectives

These works explored the evolutionary and adaptive history of ethnolinguistically different populations, drawing on the genetic diversity revealed by various genetic markers (SNPs, STRs, and others) across autosomal, Y-chromosomal, and mitochondrial DNA. These studies deepened our understanding of the broad landscape of genetic diversity and elucidated the genetic foundations of human diseases and other phenotypes. Generally, the patterns of observed genomic diversity and fine-scale population structures revealed based on autosomal and uniparental genetic markers with different densities provide new insights into the migration and admixture history of ethnolinguistically diverse populations.
